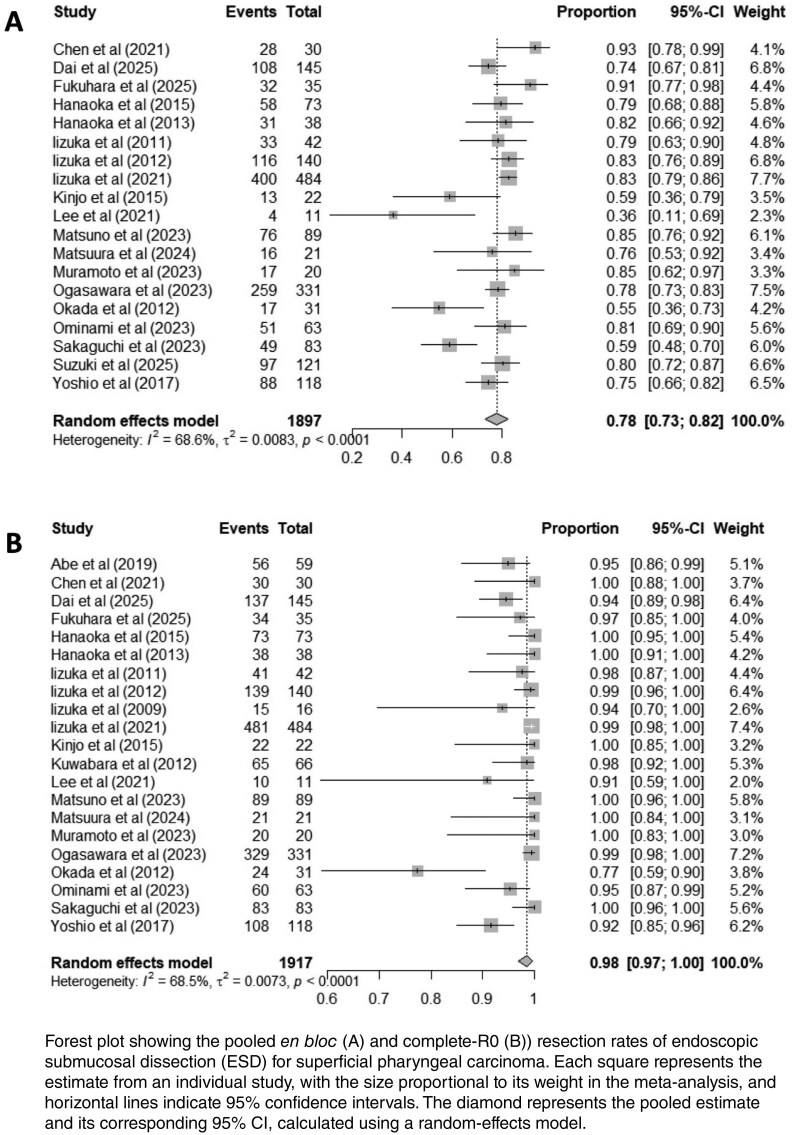# Poster Session I - A58 EFFICACY AND SAFETY OF ENDOSCOPIC SUBMUCOSAL DISSECTION FOR SUPERFICIAL PHARYNGEAL CARCINOMAS: A SYSTEMATIC REVIEW AND META-ANALYSIS

**DOI:** 10.1093/jcag/gwaf042.058

**Published:** 2026-02-13

**Authors:** G Latorre, D Kim, D Cabrera-Hinojosa, H Benites-Goñi, R Bechara

**Affiliations:** Division of Gastroenterology, Kingston Health Sciences Centre, Queen’s University, Kingston, ON, Canada; Division of Gastroenterology, Kingston Health Sciences Centre, Queen’s University, Kingston, ON, Canada; Hospital Nacional Dos de Mayo, Lima District, Lima Region, Peru; Universidad San Ignacio de Loyola, Lima, Lima Region, Peru; Division of Gastroenterology, Kingston Health Sciences Centre, Queen’s University, Kingston, ON, Canada

## Abstract

**Background:**

Endoscopic submucosal dissection (ESD) has become an important therapeutic option for superficial pharyngeal carcinoma (SPC), allowing curative resection with preservation of function. However, current evidence is largely limited to observational studies from Asian cohorts.

**Aims:**

To evaluate the efficacy and safety of ESD for SPC through a systematic review and meta-analysis.

**Methods:**

A systematic search of PubMed, Embase, and the Cochrane Library (January 2020–September 2025) identified studies reporting outcomes of ESD for SPC with ≥10 patients. Primary outcomes were *en bloc* and complete (R0) resection rates; secondary outcomes included adverse events and recurrence. Pooled estimates with 95% confidence intervals (CI) were calculated using a random-effects model, with heterogeneity assessed by I^2^. Publication bias was evaluated using Egger’s test.

**Results:**

Twenty-six observational studies (1,544 patients; 2,049 lesions) met inclusion criteria, all from Asia. Mean age was 66.3 years, 91.2% male. Most lesions arose in the hypopharynx (81.2%), with median size ranging from 5 to 23 mm. The pooled *en bloc* resection rate was 98% (95% CI: 97-100%), and the complete (R0) resection rate was 78% (95% CI: 73–82%), with substantial heterogeneity (I^2^ 68.5% and 68.5%, p < 0.0001). The pooled rates of adverse events, with and without laryngeal edema were 11% (95% CI: 2-24%) and 4% (95% CI: 2-8%), respectively. Local and lymphatic recurrence were 0.5% (95% CI: 0.0-1.6%) and 2.4% (95% CI: 1.1-4.2%), respectively. No significant publication bias was detected (p = 0.19).

**Conclusions:**

This meta-analysis demonstrates that ESD provides consistently high rates of curative resection with low recurrence for SPC across large Asian observational cohorts. While evidence from Western populations is lacking, the strength and reproducibility of current data strongly support ESD as a treatment option. Prospective multicenter studies in diverse settings are warranted to confirm generalizability.

Forest plot showing the pooled *en bloc* (A) and complete-R0 (B)) resection rates of endoscopic submucosal dissection (ESD) for superficial pharyngeal carcinoma. Each square represents the estimate from an individual study, with the size proportional to its weight in the meta-analysis, and horizontal lines indicate 95% confidence intervals. The diamond represents the pooled estimate and its corresponding 95% CI, calculated using a random-effects model.

**Funding Agencies:**

None